# Quality of Life (QoL) Survey in Hong Kong: Understanding the Importance of Housing Environment and Needs of Residents from Different Housing Sectors

**DOI:** 10.3390/ijerph15020219

**Published:** 2018-01-27

**Authors:** Zhonghua Gou, Xiaohuan Xie, Yi Lu, Maryam Khoshbakht

**Affiliations:** 1 School of Engineering and Built Environment, Griffith University, Gold Coast, QLD 4215, Australia; z.gou@griffith.edu.au (Z.G.); m.kh@griffith.edu.au (M.K.); 2 School of Architecture and Urban Planning, Shenzhen University, Shenzhen 518060, China; 3 Shenzhen Key Laboratory of Built Environment Optimization, Shenzhen University, Shenzhen 518060, China; 4 Department of Architecture and Civil Engineering, City University of Hong Kong, Hong Kong, China; yilu24@cityu.edu.hk

**Keywords:** quality of life, housing environment, liveability, housing satisfaction, housing needs, planning and design, Hong Kong

## Abstract

This study presents a Quality of Life (QoL) survey to understand the influence of the housing environment and needs of residents from different housing sectors. The research focuses on Hong Kong where living conditions have become the main affect for people’s QoL. Through a household survey using a standard instrument “Word Health Organisation (WHO) Quality of Life-BREF”, the article found that among the four WHO QoL domains (Physical Health, Psychological Health, Social Relations And Environment), Environment, particularly its constitute aspect housing environment was the most influential factor for overall quality of life for the public rental housing sector where low-income people live. This research also found that different groups of people have differing needs of their housing environments: the low-income group needs better location and privacy while the medium and high-income groups need better architectural quality. Based on differentiating their needs and wants, this research argues for prioritizing the low-income group’s needs for effectively improving their QoL.

## 1. Introduction

### 1.1. Quality of Life and Housing

Quality of Life (QoL) is an important measurement for cities’ liveability and habitability. According to WHO’s extensive definition, QoL refers to “individual’s perception of his or her position in life in the context of the culture and value systems in relation to goals, expectations, standards and concerns” [1]. For a detailed review of theoretical perspectives on QoL, Sirgy [2] classified six major theoretical concepts: socio-economic development, personal utility, just society, human development, sustainability, and functioning. Fassio, Rollero, & De Piccoli [3] categorized three main aspects for defining QoL: individual (physical and psychological health), interpersonal (social relationships) and contextual (environment) aspects. Marans [4] addressed that QoL is a multi-faceted concept and has a complex composition that defies precise definition. So, it is perhaps not surprising that there is neither an agreed upon definition nor a standard form of measurement.

In spite of there being no agreed upon definition or standard form of measurement, various organizations have attempted to rate cities and countries in terms of QoL. These attempts have tried to use different sets of characteristics to measure QoL. Kahn [5] suggested that the QoL had many dimensions including families, jobs, financial situation, health, faith, and leisure; therefore, it is a composite on an individual’s psychological and physical well-being and closely linked to concepts like satisfaction, human development, happiness, and wellness. Węziak-Białowolska [6] investigated aspects of urban quality of life in European cities and analysed the following dimensions which are potentially related to QoL: availability of services, environment and social aspects in cities and neighbourhood, socio-demographic factors; and city characteristics such as economic development, labour market pressures, size, location, quality of institutions and safety. Mercer’s Quality of Living Ranking covers 10 categories: political and social environment, economic environment, socio-cultural environment, medical and health considerations, schools and education, public services and transportation, recreation, consumer goods, housing, and natural environment [7]. However, quantifying QoL faces numerous debates, for example: what aspects should be measured and what is the relative weight of different aspects [8]. 

Recent literature on QoL has started to focus on specific contextual factors instead of general rating or weighting. These contextual factors are such as rural and urban/metropolitan areas [9,10,11] and population density [3,12,13,14]. It has been clearly shown that people’s relation to their living environments is a key issue in their quality of life [12,15,16,17,18]. Well-designed housing has been identified as an important factor in promoting quality of life [19]. Good quality housing is also instrumental in fulfilling the health and social care agendas [20,21]. Meanwhile, there is a long track of research on housing satisfaction which investigated aspects that determined occupants’ satisfaction of their housing conditions [22]. Ukoha & Beamish [23] examined the residential satisfaction with public housing in Nigeria and the relationship of satisfaction with specific housing features to overall housing satisfaction, pointing out that residents were dissatisfied with structure types, building features, housing conditions, and housing management while they were satisfied with the neighborhood facilities. Liu [24] identified key factors that had a positive correlation with residential satisfaction in Hong Kong: spatial movement within the housing, convenience of location, appropriateness of site, management and maintenance of the estate and the surroundings. Elsinga and Hoekstra [25] addressed the importance of home ownership or tenure in occupants’ satisfaction with their housing conditions, which is supported by Thomsen and Eikemo [26], Teck-Hong [27] and Herbers & Mulder [28].

### 1.2. Quality of Life in Hong Kong

Hong Kong is one of the most densely populated territories in the world. In different global cities’ ranking systems, Hong Kong is highly ranked in terms of economic prosperity while moderately or even lowly ranked in terms of QoL due to pricy housing and poor living conditions. The complexity and contradiction make Hong Kong an interesting case to explore the relationship between QoL and Housing Environment.

The local QoL Index, developed by the Faculty of Social Science of The Chinese University of Hong Kong, covers a wide range of life domains and consists of 23 indicators that are grouped into five sub-indices: Health, Social, Culture and Leisure, Economic, and Environmental [29]. The indicators are selected according to the coverage, measurability, representativeness, and importance to the quality of life in Hong Kong. The higher the score is, the better the performance. The index uses the year 2002 as the base year of the study. The index scores demonstrate that in general the quality of life in Hong Kong had improved slightly regardless of ups and downs. Specifically, the Culture & Leisure and Environmental sub-indices slightly improved, while the Health and Social sub-indices dropped in different degrees. Particularly, the Social sub-index dropped significantly. Among all sub-indicators, the housing affordability index dropped to a record low, a result indicating that housing has become continuously less affordable. Along with this, the stress index, general life satisfaction, press criticism index, government performance index, cultural programs attendance, recreation and sport activities participation, public expenditure on education, real wage index and the water index also worsened [30]. 

On the whole, despite the rapid and sustained economic development in Hong Kong over the last decades, large-scale democratic, environmental, and spiritual movements in Hong Kong did not happen, especially when the economy was in good shape. The rapidly escalating social inequality and life stresses have caused a less impressive standard of living for many people living in Hong Kong. Sing [31] examined how Hong Kong people valued and prioritized various life attributes that might affect quality of life. The study used five spheres (personal life, interpersonal life, material life, non-material life, and public life) and related 16 life domains (including housing, friendships, marriage, health, education, job and so forth). It indicated that 38% of people claimed that they rarely or never experienced enjoyment, and 44% said they had very little or no accomplishment. 59% of respondents voted “having a comfortable home” as the most prized life attribute. Obviously, the living condition has become the main affect for the quality of life in Hong Kong. This is supported by a recent QoL study in Hong Kong which found that living environment was a significant predictor of resident’s QoL [32]. Improving housing environments is of great importance to enhance QoL index for Hong Kong. To meet this end, research is needed to better understand people’s needs on QoL and related living environments. 

### 1.3. Research Objectives

The first objective of this study is to understand the role of housing environments in people’s QoL. QoL is significantly shaped by evaluation of personal lives or the place individuals live in; while the individuals’ evaluation of the place they live in is affected by the residential environmental characteristics. Although there is a long track of research about housing or residential satisfaction, few correlated the satisfaction studies with quality of life. The correlation of housing environments with QoL could help understand the importance of housing in improving cities’ habitability. There are many housing environmental characteristics including physical environments (such as ventilation, lighting and noise) and social-psychological environments (such as facilities and connections). This research also aims to find out specific housing environmental characteristics that influence QoL, which helps to inform housing planning and design decision making. 

The second objective of this study is to find out needs of residents from different housing sectors on QoL and related housing environment. In Hong Kong, there are three housing sectors: Public Rental Housing, Subsidised Sale Housing and Private Housing, which represents three different levels of economic statuses. Although local researchers had pointed out the importance of housing conditions in general quality of life and well-being, the specific housing environmental needs of different groups of residents are missing in the literature. Public Rental Housing is most vulnerable group in terms of economic statuses; therefore, their housing needs should be prioritized in housing policy and related practice. This research aims to find their needs in comparison with the needs from the other two groups, which can help inform housing policy making to improve the QoL of the low-income group.

## 2. Materials and Methods

### 2.1. Household Survey

A household survey was conducted to collect data. The survey was conducted with the help of a real estate company in Hong Kong. The strategy was to cover the three main housing sectors. So the questionnaire survey was distributed to different groups of people: from cleaners and security guards to senior managers and executives. Their responses were anonymous. There was limited influence from the company since participants’ responses would not affect their relationship with the company. Six hundred copies of questionnaires were distributed, 492 responses were returned, and 410 that were fully completed were valid for the analysis. The gender of respondents was approximately half male (50.2%) and half female (49.8%). The respondents who are below 40 years-old accounted for 59.5% of total respondents. According to Hong Kong Census and Statistics Department [33], 40 is a watershed in categorising young and old statistically. Their housing types and locations are shown in Table 1 and Table 2, respectively. The responses are well proportionate to the governmental statistics. 

Table 3, Table 4, Table 5 and Table 6 are cross-tables between demographics and housing sectors. The Chi-square (χ^2^) tests whether there are statistically significant associations between two variables. The value 0.000 indicates statistically significant associations between housing sectors and other demographic data (such as education, household income, housing size and population). Respondents living in private housing seemed to have higher education backgrounds and higher household incomes. The samples covered a large spectrum of housing size (from less than 20 square metres to more than 100 square metres). The public rental housing size in this study was mainly found between 20–59 square metres and no samples over 80 square metres. The subsidised sale housing size in this study was mainly between 40–59 square metres; neither small size (less than 20 square metres) nor large size (more than 100 square metres) was found in this study. The average housing size for the public private housing sector was larger than that for the public rental housing and subsidised sale housing sectors; the range of the housing size covered the whole spectrum from less than 20 square metres to more than 100 square metres. The public rental and subsidised sale housing sectors on average had medium population size while the private housing sector tended to have larger population size for each household. The comparison of demographics in the three housing sectors discloses the disadvantage of public rental housing where residents had lower education levels, lower incomes and less living area per person. The subsidised sale housing residents had better conditions than the public rental housing residents but poorer than the private housing residents. 

### 2.2. Survey Instrument

This survey collected residents’ opinions on two main aspects: quality of life and housing environment satisfaction. For the quality of life, the survey used a standard instrument “WHO Quality of Life-BREF” (Table 7) which contains 26 items (facets): 2 items for the overall quality of life and general health facets and 24 items which cover four domains of QoL [35]. This instrument has been used by different studies across the world. Each item is rated on a 5-point Likert scale. The use of this instrument in this household survey had been approved and authorised by WHO Information, Evidence and Research (IER) Department. The housing environment satisfaction covered 9 items (aspects): housing location, appearance, size, daylighting, natural ventilation, outdoor views, noise, privacy and layout. The nine aspects have been investigated in other studies related to housing environment in Hong Kong [36,37,38] and worldwide [39,40]. Each item is rated on a 5-point Likert scale from 1 very unsatisfied to 5 very satisfied. 

The alpha coefficient for the WHO QoL facets in this survey is 0.934, suggesting that the facets had high internal consistency and reliability. Using the WHO QoL calculation standard, raw domain scores were calculated by straightforward summative scaling of constituent items (Table 8). Three negatively-worded items were reverse-scored. Because each domain comprises a different number of items, the upper and lower possible raw score and the overall raw score differs for each domain. To be comparable, the raw domain scores were transformed to a 0–100 scales. The transformation used the following formula: transformed score = (actual raw domain score − lowest possible raw domain score) × 100/possible raw domain score range. The transformation covered the lowest possible score to 0 and the highest possible score to 100. Scores between these values represent the percentage of the total possible score achieved. 

## 3. Results

### 3.1. Four Domains of QoL

The transformed scores for WHO QoL domains are shown in Table 9. Each domain score range is 0–100. Higher scores indicate better Quality of Life. Among the four domains, Physical Health got the lowest score; Environment was the second lowest; Social Relations was the highest. The correlation analysis in Table 10 shows that the four domains are significantly correlated with each other. Among the four domains, Environment had the strongest correlations with other domains, which means that the living environment would significantly influence the other quality of life aspects. The scores on the four domains for participants under 40 were lower than participants who were 40 and above, especially on Physiological Health and Environment where statistical significance was found (*p* < 0.05). The scores on the four domains for male participants were higher than those for female participants; however, statistical significance was not found. The scores on the four domains constantly increased with the increase of education level from primary to higher education; however, no statistical significance was found. The scores also increased constantly with the increase of household income and housing size, especially on Social Relations and Environment where statistical significance was found (*p* < 0.05). No trend or statistical significance were found for household population. 

### 3.2. Housing Type and Quality of Life

Table 11 shows the scores of QoL domains for each housing sector. The highest score was found on Social Relations in the subsidized sale housing sector while the lowest score was found on Environment in the public rental housing sector. The research used Kruskal-Wallis test to compare the difference of QoL domains between each housing sector. The Kruskal-Wallis test is a non-parametric method used for comparing two or more independent samples of equal or different sample sizes. There is no significant difference on Physical Health. Residents from the private housing sector reported much higher Psychological Health than those from the public rental housing sector (*p* < 0.05). Residents from the subsidized sale housing sector and the private housing sector reported better Social Relations and Environment than those from the public housing sector (*p* < 0.05). Generally, the comparison of housing sectors distinguishes public rental housing residents as a vulnerable group in the quality of life survey. 

To find out the contribution of the four domains and their 26 facets to the general perception of quality of life, the research conducted two regression analyses to the three housing sectors (Table 12). The first model used Overall QoL as the dependent variable and 4 domains as independent variables; the second model used Overall QoL as the dependent variable and 24 constitute facets of the four domains as independent variables. For the public rental housing sector and the private housing sector, Environment seemed to be the most influential domain. Specifically, for public rental housing residents, the Environment mainly referred to living conditions (housing environment) while for the private housing residents, the Environment mainly referred to their financial conditions (money). Positive feeling was another influential factor for residents from the public housing sector, as indicated in Table 12. This is the sole non-environmental facet for the low income group. Positive feeling has an important role in shaping health and wellbeing. The increasing housing price and wealth disparity brought negative feeling to the society, which significantly affects the QoL of the low-income group who lives in the public rental housing sector. This finding indicates their psychological needs. For the subsidised sale housing sector, Physiological Health was the most influential domain; specifically, there are a number of influential items, such as working capacity, transport, and bodily appearance. 

### 3.3. Housing Environment Satisfaction

Table 13 summarises the nine housing environmental satisfactions. Among all aspects, the lowest satisfactions were found on the housing privacy, size and noise in the public rental housing sector while the highest satisfaction was found on the housing location in the private housing sector. The alpha coefficient for the nine housing environmental satisfaction items is 0.906, indicating that these items have high internal consistency and reliability. Kruskal-Wallis test was used to compare the difference between each housing sector. Significant differences were found on location, appearance, size, privacy and layout. There is no significant difference on daylighting, natural ventilation, outdoor views and noise. Specifically, on the aspect of housing location, private housing residents were more satisfied than public rental housing residents (*p* < 0.05). On the aspect of housing appearance, subsidized sale housing residents were more satisfied than public rental housing residents (*p* < 0.05). On the aspect of housing size, residents from the subsidized sale housing sector seemed to be more satisfied than those form public rental housing and private housing sectors (*p* < 0.05). On the aspect of housing privacy, private housing residents were more satisfied than subsidized housing and public rental housing residents (*p* < 0.05). On the aspect of housing layout, private housing residents were more satisfied than public rental housing residents (*p* < 0.05). 

Table 14 shows the correlation between the nine environmental aspects and four domains of WHO QoL. All environmental aspects heavily loaded on the domain of Environment. Table 15 further correlates the nine environmental aspects and the eight constitute facets of the domain of Environment. All environmental aspects heavily loaded on the facet of housing environment. The results suggest that the nine environmental aspects can help to define the housing environment that contributes to an important domain of QoL: Environment. Focusing on the housing environment, regression analyses were conducted to find out different environmental predicators for the three housing sectors (Table 16). For the public rental housing sector, the most significant predicators for the housing environment were location and privacy. For the subsidised sale housing and private housing sector, the most significant predicator was appearance. For the private housing sector, size and layout were also important predicators. This result disclosed that the private housing and subsidised rental housing residents were more concerned about psychological aspects of housing design: whether the appearance of their housing met the image of their life in the city; while the public rental housing residents were more concerned about the physical aspects of housing design, such as whether the housing is well located for accessing facilities and job opportunities. 

## 4. Summary and Discussion

First of all, the three groups of respondents, respectively from the public rental housing sector, the subsidized sale housing sector and the private housing sector, scored significantly different on the domains of WHO QoL except for Physical Health. The domain of Physical Health seemed to be a macro scale assessment of quality of life, which may be related to the government health care policy and general health status of population. The other three domains: Psychological Health, Social Relations and Environment, were scored differently among the three housing sectors, especially on the domain of Environment which refers to the specific living conditions, facilities and material life. The domain of Environment was also the most influential domain towards the general quality of life for the public rental housing and private housing groups. However, the two groups were inclined to different aspects: the public rental housing group was more concerned with living conditions while the private housing group was more concerned with the financial conditions. The subsidized sale housing residents who were considered as the medium-income group was more concerned with psychological conditions such as body appearance. The comparison implies that the improvement of the physical environment of housing design may have significant influence on the public rental housing sector while the influence is less significant on the other two housing sectors. 

Secondly, the research investigated housing satisfaction. Particularly, this research selected nine housing environmental aspects for investigation. Among all, housing location scored highly as the most satisfactory aspect in the private housing sector while housing privacy scored lowly as the most dissatisfactory aspect in the public rental housing sector. The land policy in Hong Kong gave private housing priority in well-connected locations with excellent facilities accessibility; while the other two public housing projects tended to be located in fringe of each district [41]. The high score at housing locations reflects the private housing residents’ satisfaction with this policy. The low satisfaction with privacy, size and noise in the public rental housing sector was largely due to the lack of space [36,42]. The demographic data showed that the public rental housing households tended to have small housing size and large family size. The lack of space also means a lack of privacy for individual persons inside a residential unit and also a high exposure to noise pollution from outside. 

Thirdly, the research explored how these housing environmental satisfactions influenced the domain of Environment, especially the constituent facet: housing environment. For the public rental housing, the most influential aspects were location and privacy which were also the most vulnerable aspects for this housing sector. The other two groups cared more about housing appearance when they assessed the housing environment in the WHO QoL scales. The comparison uncovered that the low-income group were still struggling for the physical condition such as location (for good accessibility to facilities) and privacy (for personal life) while that for the medium and high-income group, they started to concern the architectural quality of their housing. It is interesting to find that although many research efforts have been spent on investigating the physical conditions of housing in Hong Kong such as daylighting and ventilation [36,43], the three groups scored similarly on these aspects and these aspects had little influence on scoring the WHO QoL scale. The natural ventilation and daylighting in Hong Kong’s residential environments were notorious due to the high dense urban form and cruciform layout favorable for maximum saleable gross floor areas (GFA) [42]. The uniform market formula of producing residential floor plans made these physical environmental conditions less differentiable among the three housing sectors [37]. The low-income group individuals understand what they are lacking, such as lacking an ideal location compared to other groups, in order to achieve a better quality of life. The medium- and high-income groups, too, understand what can be improved for a better quality of life, such as better architectural quality and esthetics.

Based on the series of comparison, it is necessary to differentiate needs and wants on QoL and related housing environments. Housing environments, as basic needs, are the most influential factor for low-income group residents to assess their QoL; while they are not influential for medium- and high-income group residents who may be concerned more about appearance and material life which could be seen as their wants. In terms of specific housing satisfaction, the needs and wants are more differentiable. For example, the low-income group paid more attention to the physical conditions (such as location and privacy) which could be seen as their needs for standard living while the other two groups concerned more about architectural design quality which could be seen as their wants for aesthetic pursuits. The differentiation of needs and wants for different groups can help take effective measures in response to improving QoL. As a vulnerable group, the needs of the low-income group should be prioritized in improving QoL. In this case, their needs for housing privacy could be improved by pragmatic planning and design while their needs for better housing location may involve government policy and budget which may require long term efforts.

Besides housing environments, this research also found other aspects that differentiate the needs and wants of the people with different economical statuses. The low income group living in the public rental housing needs positive feeling to improve their QoL. The medium income group living in the subsidized housing sector needs and wants work capacity and good appearance which can bring them better opportunities to increase their economic and social statuses. The high income group living in the private housing sector wants more money to sustain their QoL in response to the increasing inflation rate. This finding inspires us to rethink the way of defining QoL in a “one-fits-all” approach and also urges us to redefine the needs and wants in improving QoL. Basic human needs should be addressed in measuring QoL.

## 5. Conclusions

Usually, quality of life is superficially understood as a high living standard or socio-economic indicator such as GDP. This study addresses individuals’ perceptions of quality of life. According to WHO, there are four important domains underlying people’s quality of life: Physical Health, Psychological Health, Social Relations and Environment. Among the four domains, Environment and its constitute facet housing environments were found in this study as the most influential factor for overall quality of life, especially for the public rental housing sector where low-income people live. In other words, the housing planning and design has an important role in enhancing their quality of life in Hong Kong. For the subsidized sale housing and private housing sectors where medium- and high-income people live, the physical housing environments seem to be less important compared to psychological aspects and material life. Based on understanding the different groups’ needs and wants, the research argues for prioritizing the low-income group’s needs for effectively improving their QoL. Specifically, their needs for better privacy could be responded to by pragmatic planning and design of buildings while their needs for better location may require long-term policy change from the government.

This study has several limitations. The first is the sample size. Although the sample could represent the three housing sectors proportionately, larger sample size is needed to further confirm the statistical significance. The second is the instrument. WHO QoL questionnaire is a well-established instrument to measure QoL; however, it is generally designed for a universal context. There should be more specific questions in response to particular contexts such as high density Asian cities where the working and living standard is different from the western society. This study calls for more research concerning QoL in different urban contexts and special concerns should be given to the low-income group to better understand their needs. 

## Figures and Tables

**Table 1:** Housing sectors.

Housing types	Responses		Government Statistics [34]
	Count	Percentage	
Public Rental Housing	112	27.3%	29.1%
Subsidised Sale Housing	80	19.5%	16.5%
Private Housing	218	53.2%	53.8%
Total	410	100%	100%

**Table 2:** Sample locations.

Locations	Responses		Government Statistics [34]
	Count	Percentage	
Hong Kong Island	75	18.3%	18.0%
Kowloon	118	28.8%	29.8%
New Territories	217	52.9%	52.2%
Total	410	100%	100%

**Table 3:** Housing sector by education level.

Housing Types	Education (χ^2^ = 0.000)			
	Primary Education	Secondary Education	Tertiary and Higher Education	Total
Public Rental Housing	16%	45%	39%	100%
Public Rental Housing	4%	51%	45%	100%
Private Housing	4%	29%	67%	100%

**Table 4:** Housing sector by household income.

Housing Types	Household Income (HK$) (χ^2^ = 0.000)				
	<10,000	10,000–20,000	20,000–40,000	>40,000	Total
Public Rental Housing	12%	40%	39%	9%	100%
Subsidised Sale Housing	5%	36%	42%	17%	100%
Private Housing	4%	20%	46%	30%	100%

**Table 5:** Housing sector by housing size.

Housing Types	Housing Size (m^2^) (χ^2^ = 0.000)						
	<20	20–39	40–59	60–79	80–99	>100	Total
Public Rental Housing	8%	32%	39%	11%	0%	0%	90% (10% missing)
Subsidised Sale Housing	0%	18%	48%	25%	9%	0%	100%
Private Housing	1%	4%	30%	48%	9%	8%	100%

**Table 6:** Housing sector by household population.

Housing Types	Household Population (χ^2^ = 0.000)							
	1	2	3	4	5	6	>6	Total
Public Rental Housing	6%	15%	26%	37%	12%	3%	1%	100%
Subsidised Sale Housing	5%	15%	41%	25%	7%	7%	0%	100%
Private Housing	1%	4%	10%	20%	22%	27%	16%	100%

**Table 7:** Domains and facets of the WHO Quality of Life.

Domain	Facet
Overall QoL and General Health	Overall QoL
	Satisfaction with health
Domain1: PHYSICAL HEALTH	Physical Pain Medical Needs Enough Energy Physical Activity Sleep Quality Living Capacity Working Capacity
Domain2: PSYCHOLOGICAL HEALTH	Happiness and Enjoyment Positive Feeling Concentration Bodily Appearance Self-Satisfaction Negative Feeling
Domain3: SOCIAL RELATIONS	Personal Relations Family Life Social Support
Domain4: ENVIRONMENT	Safety Environmental Health Financial Resources Information Resources Leisure Facilities Housing Environment Healthcare Facilities Transport Facilities

**Table 8:** The equations for calculating WHO QoL domain scores.

Domain	Equations for Computing Domain Scores	Lower Value	Upper Value	Score Range
Physical Health	(6-Physical Pain) + (6-Medical Needs) + Enough Energy + Physical Activity + Sleep Quality + Living Capacity + Working Capacity	7	35	28
Psychological Health	Happiness and Enjoyment + Positive Feeling + Concentration + Bodily Appearance + Self Satisfaction + (6-Negative Feeling)	6	30	24
Social Relations	Personal Relations + Family Life + Social Support	3	15	12
Environment	Safety + Environmental Health + Financial Resources + Information Resources + Leisure Facilities + Housing Environment + Healthcare Facilities + Transport Facilities	8	40	32

**Table 9:** The WHO QoL domains.

Domains	Mean	Standard Deviation	Min.	Max.
Physical Health	60.3	13.9	7	100
Psychological Health	62.7	15.8	13	100
Social Relations	65.9	17.9	8	100
Environment	60.9	16.8	6	100

**Table 10:** Correlation table.

Domains		Physical Health	Psychological Health	Social Relations
Psychological Health	Pearson Correlation	0.660 **		
	Sig. (2-tailed)	0.000		
Social Relations	Pearson Correlation	0.550 **	0.696 **	
	Sig. (2-tailed)	0.000	0.000	
Environment	Pearson Correlation	0.664 **	0.750 **	0.692 **
	Sig. (2-tailed)	0.000	0.000	0.000

** Correlation is significant at the 0.01 level (2-tailed).

**Table 11:** Quality of life domains and housing sectors.

Quality of Life Domains	Housing Types	Mean	Standard Deviation	Min.	Max.
Physical Health	Public Rental Housing	59.25	13.889	14	93
	Subsidised Sale Housing	59.69	14.299	25	93
	Private Housing	61.12	13.970	7	100
Psychological Health	Public Rental Housing	58.98	14.849	13	100
	Subsidised Sale Housing	63.44	18.132	21	100
	Private Housing	64.45	15.280	0	100
Social Relations	Public Rental Housing	61.44	19.119	0	100
	Subsidised Sale Housing	67.92	19.537	25	100
	Private Housing	67.55	16.264	8	100
Environment	Public Rental Housing	56.65	15.421	6	100
	Subsidised Sale Housing	63.28	17.520	25	100
	Private Housing	62.24	16.940	0	100

**Table 12:** Predicators for the overall quality of life.

Models	Quality of Life (Dependent Variable)		
	Public Rental Housing	Subsidised Sale Housing	Private Housing
Model 1: 4 domains as independent variables	Environment ** R^2^ = 0.392	Psychological Health ** R^2^ = 0.449	Environment ** R^2^ = 0.477
Model 2: 24 facets as independent variables	Housing Environment ** Positive Feeling ** R^2^ = 0.697	Working Capacity ** Transport Facilities ** Bodily Appearance ** R^2^ = 0.745	Money * R^2^ = 0.516

The beta value is a measure of how strongly each predictor variable influences the criterion (dependent) variable. ** indicates *p* < 0.01; * indicates *p* < 0.05. R^2^ is a statistical measure of how close the data are to the fitted regression model.

**Table 13:** Housing environmental satisfaction and housing sectors.

Item	Housing Sector	Mean	Standard Deviation	Min.	Max.
Location	Public Rental Housing	3.52	0.980	1	5
	Subsidised Sale Housing	3.76	0.860	1	5
	Private Housing	3.80	0.835	1	5
Appearance	Public Rental Housing	3.28	0.946	1	5
	Subsidised Sale Housing	3.65	0.797	2	5
	Private Housing	3.55	0.917	1	5
Size	Public Rental Housing	3.17	0.999	1	5
	Subsidised Sale Housing	3.59	0.837	2	5
	Private Housing	3.45	0.926	1	5
Daylighting	Public Rental Housing	3.33	1.030	1	5
	Subsidised Sale Housing	3.57	0.943	1	5
	Private Housing	3.52	0.913	1	5
Natural Ventilation	Public Rental Housing	3.45	0.932	1	5
	Subsidised Sale Housing	3.60	0.936	1	5
	Private Housing	3.49	0.975	1	5
Outdoor Views	Public Rental Housing	3.44	0.931	1	5
	Subsidised Sale Housing	3.49	0.972	1	5
	Private Housing	3.44	0.997	1	5
Noise	Public Rental Housing	3.16	0.987	1	5
	Subsidised Sale Housing	3.25	1.061	1	5
	Private Housing	3.28	1.025	1	5
Privacy	Public Rental Housing	3.07	1.024	1	5
	Subsidised Sale Housing	3.39	0.907	1	5
	Private Housing	3.44	0.950	1	5
Layout	Public Rental Housing	3.28	0.955	1	5
	Subsidised Sale Housing	3.48	0.826	1	5
	Private Housing	3.51	0.842	1	5

**Table 14:** Correlation table for the housing environmental satisfaction and four domains of quality of life.

Housing Satisfactions		Physical Health	Psychological Health	Social Relations	Environment
Location	Pearson Correlation	0.362 **	0.358 **	0.352 **	0.427 **
	Sig. (2-tailed)	0.000	0.000	0.000	0.000
Appearance	Pearson Correlation	0.316 **	0.378 **	0.350 **	0.468 **
	Sig. (2-tailed)	0.000	0.000	0.000	0.000
Size	Pearson Correlation	0.271 **	0.354 **	0.323 **	0.449 **
	Sig. (2-tailed)	0.000	0.000	0.000	0.000
Daylighting	Pearson Correlation	0.270 **	0.324 **	0.316 **	0.401 **
	Sig. (2-tailed)	0.000	0.000	0.000	0.000
Ventilation	Pearson Correlation	0.227 **	0.281 **	0.282 **	0.353 **
	Sig. (2-tailed)	0.000	0.000	0.000	0.000
View	Pearson Correlation	0.240 **	0.295 **	0.283 **	0.390 **
	Sig. (2-tailed)	0.000	0.000	0.000	0.000
Noise	Pearson Correlation	0.192 **	0.296 **	0.303 **	0.356 **
	Sig. (2-tailed)	0.000	0.000	0.000	0.000
Privacy	Pearson Correlation	0.226 **	0.389 **	0.323 **	0.439 **
	Sig. (2-tailed)	0.000	0.000	0.000	0.000
Layout	Pearson Correlation	0.289 **	0.336 **	0.306 **	0.441 **
	Sig. (2-tailed)	0.000	0.000	0.000	0.000

** Correlation is significant at the 0.01 level (2-tailed).

**Table 15:** Correlation table for the housing environmental satisfaction and the domain of environment.

Housing Satisfactions		Safety	Environmental Health	Financial Resources	Information Resources	Leisure Facilities	Housing Environment	Healthcare Facilities	Transport Facilities
Location	Pearson Correlation	0.392 **	0.347 **	0.229 **	0.279 **	0.305 **	0.395 **	0.351 **	0.304 **
	Sig. (2-tailed)	0.000	0.000	0.000	0.000	0.000	0.000	0.000	0.000
Appearance	Pearson Correlation	0.321 **	0.350 **	0.316 **	0.359 **	0.297 **	0.441 **	0.400 **	0.357 **
	Sig. (2-tailed)	0.000	0.000	0.000	0.000	0.000	0.000	0.000	0.000
Size	Pearson Correlation	0.270 **	0.362 **	0.349 **	0.324 **	0.265 **	0.448 **	0.326 **	0.345 **
	Sig. (2-tailed)	0.000	0.000	0.000	0.000	0.000	0.000	0.000	0.000
Daylighting	Pearson Correlation	0.199 **	0.363 **	0.339 **	0.307 **	0.251 **	0.380 **	0.308 **	0.274 **
	Sig. (2-tailed)	0.000	0.000	0.000	0.000	0.000	0.000	0.000	0.000
Ventilation	Pearson Correlation	0.160 **	0.337 **	0.281 **	0.248 **	0.213 **	0.372 **	0.289 **	0.239 **
	Sig. (2-tailed)	0.001	0.000	0.000	0.000	0.000	0.000	0.000	0.000
View	Pearson Correlation	0.209 **	0.326 **	0.283 **	0.268 **	0.304 **	0.376 **	0.287 **	0.301 **
	Sig. (2-tailed)	0.000	0.000	0.000	0.000	0.000	0.000	0.000	0.000
Noise	Pearson Correlation	0.200 **	0.278 **	0.274 **	0.245 **	0.232 **	0.335 **	0.292 **	0.268 **
	Sig. (2-tailed)	0.000	0.000	0.000	0.000	0.000	0.000	0.000	0.000
Privacy	Pearson Correlation	0.249 **	0.350 **	0.360 **	0.330 **	0.264 **	0.416 **	0.316 **	0.355 **
	Sig. (2-tailed)	0.000	0.000	0.000	0.000	0.000	0.000	0.000	0.000
Layout	Pearson Correlation	0.270 **	0.335 **	0.373 **	0.388 **	0.292 **	0.422 **	0.338 **	0.260 **
	Sig. (2-tailed)	0.000	0.000	0.000	0.000	0.000	0.000	0.000	0.000

** Correlation is significant at the 0.01 level (2-tailed).

**Table 16:** Predicators for housing environment.

Housing Satisfactions	Housing Environment		
	Public Rental Housing	Subsidised Housing	Private Housing
Location	0.405 **	0.088	0.006
Appearance	−0.137	0.432 **	0.257 **
Size	−0.146	0.048	0.205 *
Daylighting	0.155	−0.229	−0.071
Ventilation	0.021	0.134	0.127
View	−0.055	0.132	−0.054
Noise	0.102	−0.182	−0.013
Privacy	0.324 **	0.284	0.106
Layout	0.020	0.111	0.184 *
R^2^	0.280	0.333	0.378

The beta value is a measure of how strongly each predictor variable influences the criterion (dependent) variable. ** indicates *p* < 0.01; * indicates *p* < 0.05.
